# Equity-Driven Sensing System for Measuring Skin Tone–Calibrated Peripheral Blood Oxygen Saturation (OptoBeat): Development, Design, and Evaluation Study

**DOI:** 10.2196/34934

**Published:** 2022-04-22

**Authors:** Alexander T Adams, Ilan Mandel, Yixuan Gao, Bryan W Heckman, Rajalakshmi Nandakumar, Tanzeem Choudhury

**Affiliations:** 1 Information Science Cornell Tech New York, NY United States; 2 The Center for the Study of Social Determinants of Health Meharry Medical College Nashville, TN United States; 3 Psychiatry and Behavioral Sciences, School of Medicine Meharry Medical College Nashville, TN United States; 4 Division of Public Health, School of Graduate Studies and Research Meharry Medical College Nashville, TN United States

**Keywords:** mobile health, ubiquitous health, health equity, bias, pulse oximetry, oximetry, mHealth, health app, skin tone, oximeter, smartphone, sensor, heart rate, oxygen level, oxygen saturation, pulse, mobile phone

## Abstract

**Background:**

Many commodity pulse oximeters are insufficiently calibrated for patients with darker skin. We demonstrate a quantitative measurement of this disparity in peripheral blood oxygen saturation (SpO_2_) with a controlled experiment. To mitigate this, we present OptoBeat, an ultra–low-cost smartphone-based optical sensing system that captures SpO_2_ and heart rate while calibrating for differences in skin tone. Our sensing system can be constructed from commodity components and 3D-printed clips for approximately US $1. In our experiments, we demonstrate the efficacy of the OptoBeat system, which can measure SpO_2_ within 1% of the ground truth in levels as low as 75%.

**Objective:**

The objective of this work is to test the following hypotheses and implement an ultra–low-cost smartphone adapter to measure SpO_2_: skin tone has a significant effect on pulse oximeter measurements (hypothesis 1), images of skin tone can be used to calibrate pulse oximeter error (hypothesis 2), and SpO_2_ can be measured with a smartphone camera using the screen as a light source (hypothesis 3).

**Methods:**

Synthetic skin with the same optical properties as human skin was used in ex vivo experiments. A skin tone scale was placed in images for calibration and ground truth. To achieve a wide range of SpO_2_ for measurement, we reoxygenated sheep blood and pumped it through synthetic arteries. A custom optical system was connected from the smartphone screen (flashing red and blue) to the analyte and into the phone’s camera for measurement.

**Results:**

The 3 skin tones were accurately classified according to the Fitzpatrick scale as types 2, 3, and 5. Classification was performed using the Euclidean distance between the measured red, green, and blue values. Traditional pulse oximeter measurements (n=2000) showed significant differences between skin tones in both alternating current and direct current measurements using ANOVA (direct current: *F*_2,5997_=3.1170 × 10^5^, *P*<.01; alternating current: *F*_2,5997_=8.07 × 10^6^, *P*<.01). Continuous SpO_2_ measurements (n=400; 10-second samples, 67 minutes total) from 95% to 75% were captured using OptoBeat in an ex vivo experiment. The accuracy was measured to be within 1% of the ground truth via quadratic support vector machine regression and 10-fold cross-validation (*R*^2^=0.97, root mean square error=0.7, mean square error=0.49, and mean absolute error=0.5). In the human-participant proof-of-concept experiment (N=3; samples=3 × N, duration=20-30 seconds per sample), SpO_2_ measurements were accurate to within 0.5% of the ground truth, and pulse rate measurements were accurate to within 1.7% of the ground truth.

**Conclusions:**

In this work, we demonstrate that skin tone has a significant effect on SpO_2_ measurements and the design and evaluation of OptoBeat. The ultra-low-cost OptoBeat system enables smartphones to classify skin tone for calibration, reliably measure SpO_2_ as low as 75%, and normalize to avoid skin tone–based bias.

## Introduction

### Background

The measurement of blood oxygenation is necessary in acute medical emergencies and useful for tracking physical fitness [1] and shortening recovery times [2]. Blood oxygen saturation is an essential physiological signal that can be measured noninvasively optically or invasively via blood gas measurements. Peripheral blood oxygen saturation (SpO_2_) measures the ratio of oxygen-saturated hemoglobin in the blood to hemoglobin not saturated with oxygen. Oxygen saturation levels <90% can result in a variety of symptoms and adverse health conditions.

Researchers have persistently documented how common pulse oximeters overestimate blood oxygen levels in patients with darker skin tones [3-6]. Commercial devices consistently overestimate oxygen saturation by as much as 2%, which could result in mistreated hypoxia or inadequate treatment. This paper focuses on the development of an ultra–low-cost, novel method for the measurement of SpO_2_ that can be calibrated according to an individual’s skin tone using a smartphone.

The COVID-19 pandemic has resulted in acute global shortages of necessary medical supplies, including pulse oximeters [7]. The “Silent Hypoxia” of patients with COVID-19 [8] increased the necessity for regular screening of blood oxygen levels, thus greatly increasing the demand for cheap and easily manufacturable pulse oximeters. Disruptions in supply chains were persistent [9], particularly in the silicon and chip markets [10] necessary for the construction of many medical devices. Our method makes regular pulse oximetry screenings possible for billions of pre-existing smartphone owners at a fraction of the cost of consumer pulse oximeters. Using a phone’s screen and camera to measure the divergent absorption rates of blue and red light in hemoglobin, we can measure blood oxygen levels without any additional electronics. The system can be made using approximately US $1 of commodity plastics and a plastic ear clip, which can be easily 3D-printed or cheaply manufactured at scale.

The proliferation of smartphones over the past decade provides a platform upon which a variety of health apps can be built. The combination of high-quality sensors, increasingly powerful mobile computation, and internet connectivity enables the creation of medical sensing systems that already live in billions of users’ pockets. More than 1 million mobile health apps are available on major mobile platforms [11]. This work builds on the subset of methods that use mobile phones to cheaply monitor and measure cardiovascular health. Using the camera built into most smartphones, we can calibrate for variance in skin tone that is not sufficiently captured by commodity pulse oximeters.

In this paper, we introduce the equity-driven design of OptoBeat, a novel, ultra–low-cost smartphone-based pulse oximeter. OptoBeat can determine a coefficient to normalize pulse oximetry readings for differences in skin tone. OptoBeat can measure SpO_2_ within 1% accuracy of the gold standard. We validated OptoBeat in blood oxygen levels that ranged from healthy (95%-100%) to critical (as low as 75%), corresponding to hypoxia. In this paper, we prove the following three hypotheses: (1) skin tone has a significant effect on pulse oximeter measurements (hypothesis 1), (2) images of skin tone can be used to calibrate for pulse oximeter error (hypothesis 2), and (3) SpO_2_ can be measured with a smartphone camera using the screen as a light source (hypothesis 3).

Through quantitative analysis, we demonstrate how skin tone affects pulse oximeters. We show that the unilateral effect of skin tone not only decreases the signal to noise ratio but also affects the ratio of ratios between the 2 sources, which is used to calculate SpO_2_. We further demonstrate how this can be done with a smartphone and describe the design, development, and testing of an ultra–low-cost smartphone-based pulse oximeter. OptoBeat enables blood oxygen saturation monitoring by augmenting the smartphone’s camera system, focusing the light source, and leveraging extant computing capacity. With this system, we can use a skin tone measurement to adjust the ratios of the 2 source frequencies transmitted through the skin to calibrate the measurement of blood oxygen for a more accurate reading.

Specifically, we present the following contributions: (1) an experiment and quantitative analysis of how skin tone affects traditional pulse oximeters and a demonstration of how this can be remedied; (2) the design of the OptoBeat optical sensing system and an experiment to validate the theory behind our design; and (3) the design, execution, and results from an ex vivo experiment that validates the accuracy of pulse oximetry from healthy and critical SpO_2_ levels against the gold standard and the results of a human-participant experiment to validate the efficacy of the device.

### Prior Work

#### Overview

**Figure 1:**
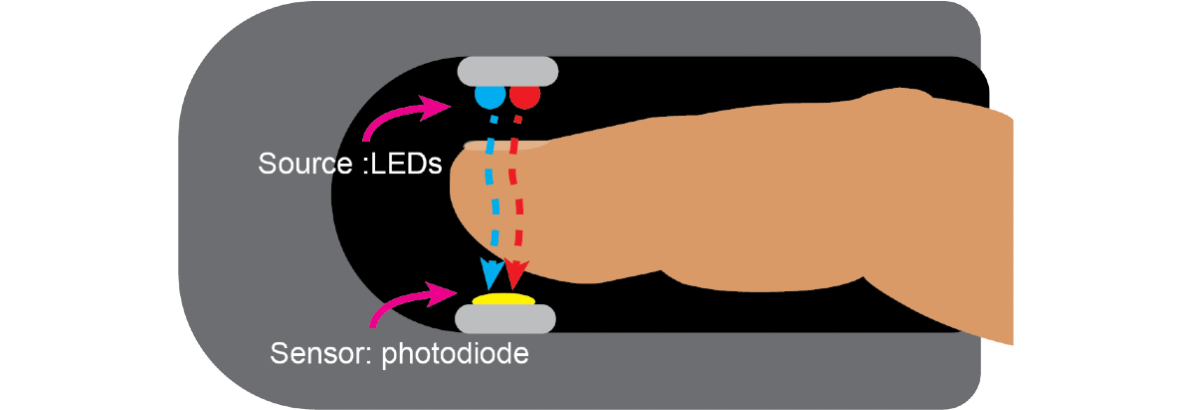
Traditional pulse oximeter design.

**Figure 2:**
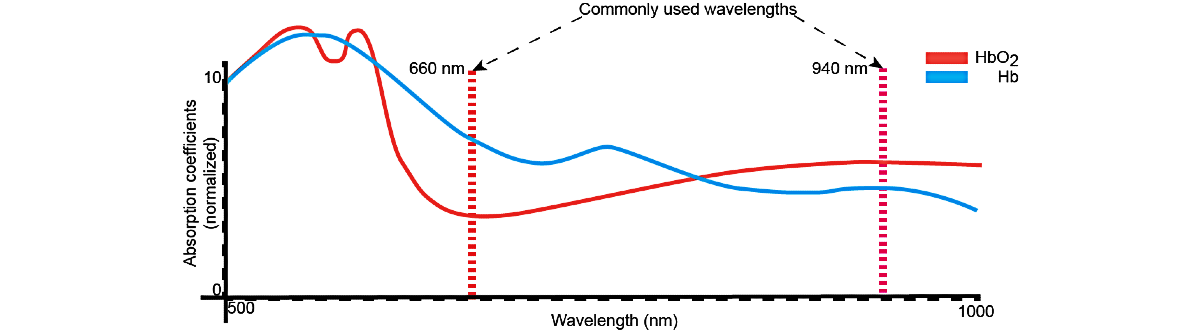
Absorption spectrum of oxygenated and deoxygenated hemoglobin and common wavelengths used in pulse oximetry. Hb: hemoglobin; HbO_2_: oxygenated hemoglobin.

Typically, pulse oximeters measure the ratio of oxygenated to deoxygenated hemoglobin through capillaries in the finger, as shown in Figure 1. To capture SpO_2_, a pulse oximeter uses at least two different electromagnetic wave sources at different wavelengths—one with a higher transmission in oxygenated hemoglobin and one with a higher transmission in deoxygenated hemoglobin, as shown in Figure 2. The ratio of the 2 wavelengths is used to determine the blood oxygen saturation [12]. Common sources used in commercial pulse oximeters are red light at approximately 660 nm and infrared (IR) light between 880 and 940 nm. This is due to both cost and the characteristics of the waves as larger wavelengths in the visible and near-IR spectrum transmit better through the skin [13].

#### Pulse Oximetry and Skin Tone

Pulse oximetry measurements using standard commercial hardware can present biases and errors. Signal quality has been shown to decrease and increase with temperature when using finger-based measurements [14]. In the study by Nowara et al [15], the authors analyzed >400 videos of 73 individuals and found significant differences in the signal to noise ratio of those with darker skin tones. Sex is also predictive of errors in pulse oxygen measurements, resulting in higher SpO_2_ estimates in women [16].

At saturation >80%, errors because of skin tone were not found to be significant; however, “in individuals with darkly pigmented skin, bias of up to 8% was observed at lower saturations” [17]. Anecdotal data showing this disparity have been documented as early as the late 1980s [17-19]. Analyses performed on patients from 2014 to 2015 and in 2020 demonstrated that “Black patients had nearly three times the frequency of acute hypoxemia that was not detected by pulse oximetry as White patients” [20]. Pulse oximeters are calibrated using correlation factors obtained by comparing the device being tested with a direct measurement of arterial hemoglobin saturation. The systematic absence of darker skinned participants during calibration may have resulted in devices optimized for whiter skin tones. Authors of a study stated the following:

In our 20 yr [sic] of testing pulse oximeter accuracy, and probably in other testing laboratories, the majority of subjects have been light skinned. Most pulse oximeters have probably been calibrated using light-skinned individuals, with the assumption that skin pigment does not matter.[16]

This is similarly backed up by the 2013 Food and Drug Administration guidance on device calibration, which states that the “study should have subjects with a range of skin pigmentation, including at least 2 darkly pigmented subjects or 15% of your subject pool” [21]. Calibrating devices using only 15% of participants with darker skin is likely an underestimation of the percentage of Americans with higher levels of skin pigmentation [22]. The failures of federal standards and their resultant measurement errors fit within the well-documented ways in which racism is encoded in medical science, research, and treatment [23-25]. The disparities in measurement have become more pressing in the face of COVID-19, a disease in which morbidity disproportionately affects Black people [26] and exhibits an unusual form of silent hypoxemia [27].

Skin tone presents common complications in both dermal and transdermal optical measurements, particularly those in the visible spectrum. To help with this issue, researchers have found it useful to have a reference. One such standard reference is the Fitzpatrick skin tone scale. Although it is not without its own racial limitations [28], we found that it provided sufficient variation for understanding how pulse oximeters can fail in patients with darker skin tones.

Previous work has demonstrated how the Fitzpatrick scale was used to calibrate video-based heart rate monitoring in people with acute hypoxia [1]. Capturing individual differences in skin tone allows for the calibration of measurement bias in ways that are not possible with traditional pulse oximeters.

#### Smartphone Pulse Oximetry

The rapid development of smartphone sensors has enabled the design and implementation of smartphone-based medical and health-sensing systems. Systems that measure cardiac signals and blood composition, including heart rate [29,30], electrocardiogram [31,32], and hemoglobin levels [33], have been developed and deployed.

Various apps have been developed in the past decade for mobile phone–based pulse oximeters [34-37]. Smartphone-based pulse oximeters typically use the phone’s flash as a light source and the rear-facing camera as a receiver. The camera has a red, green, and blue (RGB) filter over each pixel; the captured photoplethysmogram is broken down into blue and red light and then analyzed for SpO_2_ [37]. However, the phone’s flash is designed to transmit bursts of bright white light and is not stable, which would require a constant current source driver circuit for the flash’s light-emitting diode (LED). There is a substantial imbalance between red and blue light that must be accounted for. Instability may arise from small shifts in the spectral emission pattern of the LED, the wide-range wavelength captured by the blue filter, and the flash’s power supply [38]. In healthy patients, the performance of these systems [39] is in line with that of clinical pulse oximeters [40]. However, these systems perform poorly in unhealthy patients, particularly when their SpO_2_ drops below 95% [40].

Commercial smartphone apps lack detailed information on the underlying algorithms and analyses used [3,41]. Furthermore, these systems are nonconcordant with ground truth measurements and regularly fail to detect hypoxia [42]. Researchers have experimented with modifying smartphones with optical filters as well, which has shown better results but still does not have sufficient accuracy outside of healthy SpO_2_ levels [43].

Alternative smartphone-based systems incorporate an external sensor using the phone as a data hub. Many are standard fingertip-style pulse oximeters [36] with additional communication hardware such as Bluetooth Low Energy or Wi-Fi. Other smartphone-enabled form factors that are commercially available are wrist-worn and ring-based pulse oximeters [44]. Supplemental hardware can increase the accuracy of SpO_2_ measurements with costs ranging from US $30 to US $400.

### Theory: Blue Light Pulse Oximetry

We validated the electromagnetic spectrum emitted by the screen (white, all LEDs at full brightness) using a spectrometer [45]. The center (peak) band of the RGB LEDs in the screen for blue (465 nm) provided the required inverse relationship to hemoglobin that the center band of red (620 nm) has. Theoretically, this means that we can measure blood oxygen saturation with the LEDs built into a phone’s screen.

## Methods

### Overview

The OptoBeat system has several components to it, each of which we tested individually in a series of ex vivo experiments. These experiments allowed for control that was otherwise extremely difficult or impossible in a human-participant trial. For example, it would be extremely difficult to isolate skin from other components of the body (ie, blood, bone, and fat) in vivo. It is also dangerous for a healthy individual’s blood oxygen saturation to fall below 95%. To test the range of oxygen saturation required to satisfy the needs of the OptoBeat system, we had to control the full range of blood oxygen saturation required for medical diagnosis. In this section, we describe these experiments in detail as well as the design of the OptoBeat system and how it was used to test our three hypotheses (hypotheses 1-3).

### The OptoBeat Optical System

**Figure 3:**
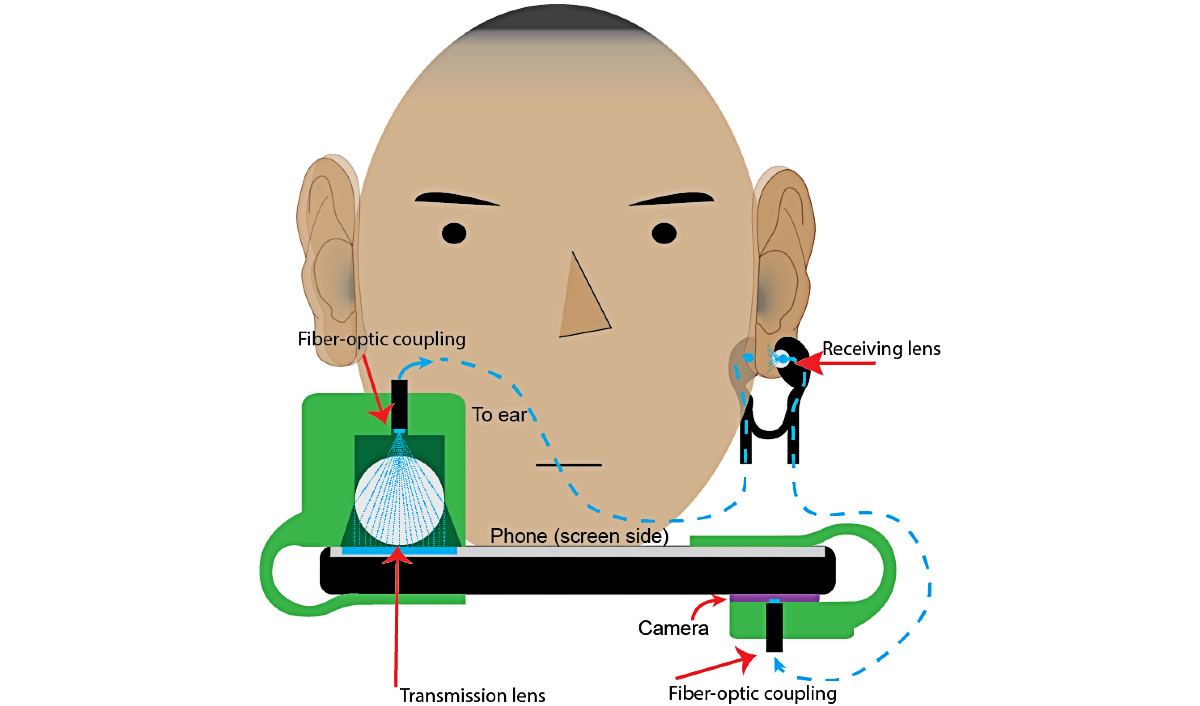
Cross-sectional diagram of the OptoBeat system.

The final design consists of three 3D-printed plastic clips, two 3-foot fiber-optic cables, 1 acrylic ball lens, and some rubber bands or O-rings for grip. The total cost is <US $1 to put together individually; if scaled up, it would likely drop in price significantly. Figure 3 shows a cross-sectional diagram of how it all fits together with the smartphone.

Our goal was to focus as much light as possible and then couple the system with fiber-optic cables to maintain signal strength and block ambient light from entering the source (screen) or the receiver (camera). We evaluated dozens of commercial optical tools and systems to obtain an idea of what had been done; how we could move light around; and what materials were readily available, machinable, moldable, and affordable. This included optical filters, gratings, mirrors, and fiber plates, among others, that ranged from US $100 for individual components to tens of thousands of dollars.

### Fabrication

We limited our design exploration to materials and fabrication techniques such that the system could be built without specialized equipment. Most of the design, testing, and fabrication was executed using a laser cutter, a 3D printer, diamond files, and a drill press. In addition, to make an affordable, robust optical system, we explored a variety of raw materials. We found that the optimal shape for capturing light and coupling it with the fiber-optic cable was a long cone with a convex lens at the base. This design both collimated the beam and acted as a wave guide for coupling the light.

To make the lens, we experimented with hand-cutting a cast acrylic rod with diamond files and then polishing them to reach optical clarity. We also made a lens with a stereolithography 3D printer using clear resin that was then dipped in resin and cured to fill layer-height artifacts. In addition, we cast the negative of a lens (using a variety of materials) and then molded it with an optically clear urethane resin. Lens construction methods have proven to be labor-intensive, delicate tasks. Looking for alternative solutions to handmade or custom lenses, we found that a spherical lens worked nearly as well. This was mostly because focal length was not of concern as the distance to the fiber-optic cable could be easily adjusted. Cheap clear acrylic spheres are easily accessible from a variety of vendors and work well enough to focus light for transmissive pulse oximeters.

### Placement

Given the limited brightness of the screen, we looked for other areas of the body in addition to the fingertip to capture SpO_2_. Our search focused on areas that were physically accessible, with a short optical path (distance between transmitter and receiver) and a high density of capillaries to improve the pulsatile signal. Three plausible solutions were the fingertip (same as traditional pulse oximeters), the webbing between the fingers, and the earlobe. Data were captured at each location, with the earlobe showing the highest signal to noise ratio for output magnitude and pulsatile signal.

The earlobe is comfortable, and a sensor could easily be worn for prolonged periods without impeding the user in most activities (no more than a set of wired headphones would), affording more continuous monitoring.

### Mobile App

**Figure 4:**
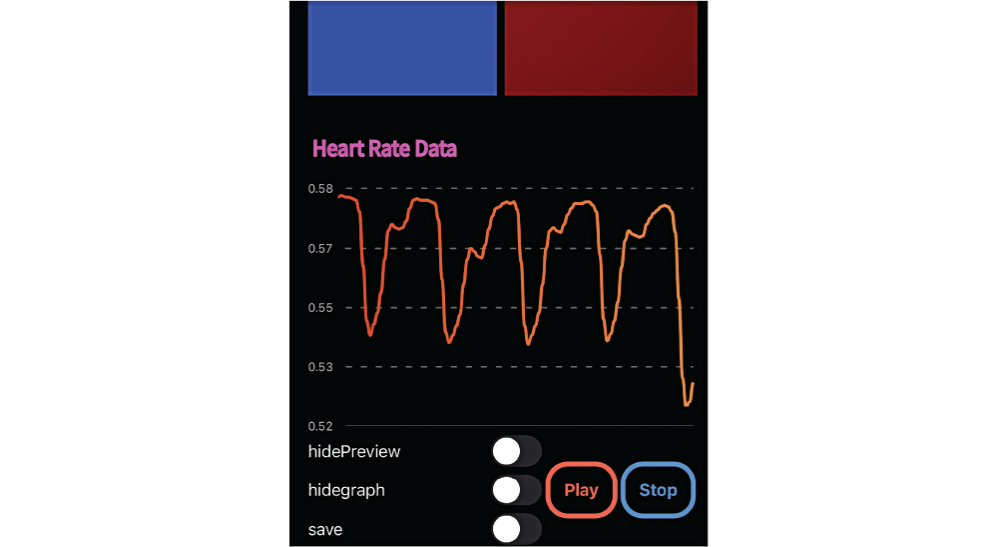
The OptoBeat mobile app.

The OptoBeat signal acquisition app was designed on the iOS platform using Swift. The data acquisition page is shown in Figure 4. The app consists of the following components: data labels, camera selection, frame rate, sensitivity (ISO), exposure time, data visualization, pulse rate, and color selection. The app captures the light that has been emitted from the screen and passed through the user’s skin. The mean values for RGB are stored on the phone for postanalysis.

Synchronizing the screen refresh rate and camera capture rate is crucial. The app ensures that the frequencies of data capture (camera) and source transmission (screen flashing) are synchronized. Misalignment results in the loss of essential information in the signal. An example of synchronization issues that we found is mixed-pulse capture. This occurs when half of the frame captures one color and the second half captures another. In our case, this was blue and red, resulting in a purple frame, which is unusable.

OptoBeat controls the camera using the Apple AVFoundation application programming interface. All automatic camera options are turned off, including autofocus, automatic white balance, and low light boost. The app allows the experimenter to control all other settings, including ISO, exposure time, and camera capture rate. The camera runs on a separate thread, and each frame of the video is sent immediately to the camera buffer. Data are directly accessed through the CMSampleBuffer (Apple Inc), and the RGB values are logged to the back end using a data management pipeline in a new thread without slowing down the main process.

### Skin Tone Experiment

**Figure 5:**
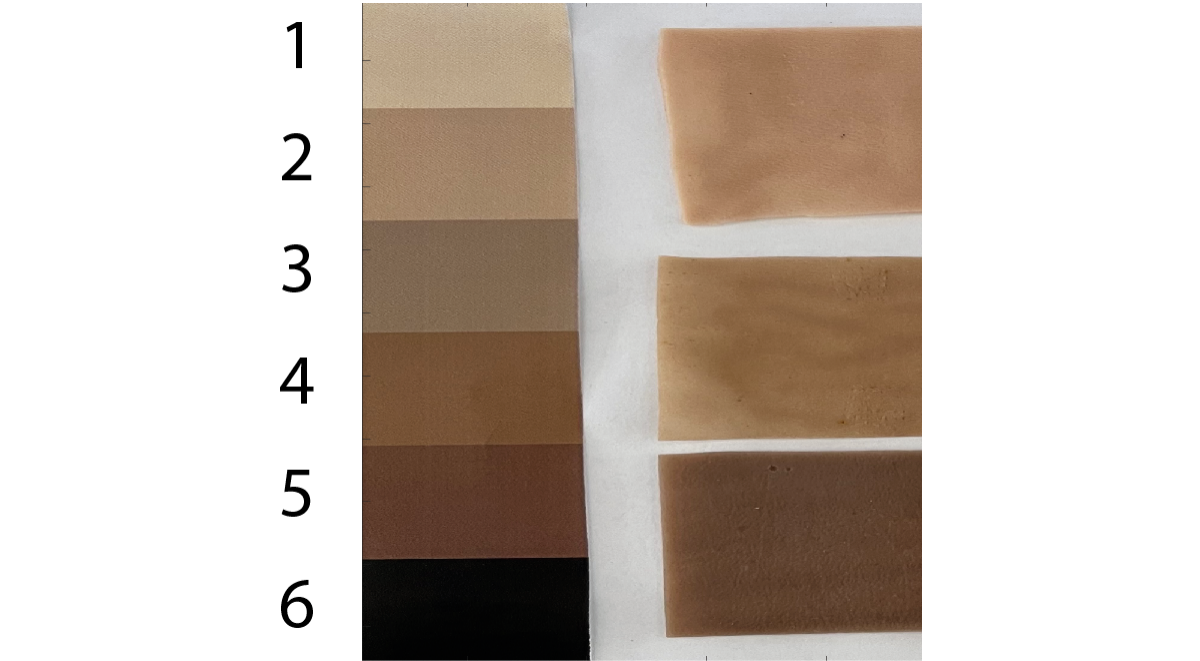
Image of skin tone calibration strip and synthetic skin.

As the behavior of electromagnetic waves in different skin tones is not necessarily continuous, we decided to classify instead of regress across the spectrum of skin. To demonstrate hypothesis 1 and test hypothesis 2, we classified the different tones as 1-6 according to the Fitzpatrick scale. For a ground truth, we printed a copy of the scale to use as a reference and placed it beside the synthetic skin [46] being tested (as shown in Figure 5) to account for variation in ambient lighting. Similar techniques have been used to measure bilirubin in the skin tone of infants to detect jaundice [47]. It should also be noted that synthetic skin is only available in 3 different tones; otherwise, we would have tested on more types.

The ground truth was determined by capturing the mean RGB value of each reference color. For each synthetic skin sample, the mean RGB was calculated and iteratively compared with the 6 ground truth classes using Euclidean distance and the absolute difference in luminance-weighted grayscale (Multimedia Appendix 1).

The results were identical for both grayscale and RGB; an example image is shown in Figure 5. Skin samples were matched to their corresponding groups on the Fitzpatrick scale in several images. The results were confirmed through visual inspection. The lighter sample matched group 2, the tan sample matched group 3, and the darker sample matched group 5.

### Blood Oxygen Saturation Experiment

After validating that we had obtained an optical signal from the earlobe through the video recording analysis of our system, we designed an experiment to test hypothesis 3, validating OptoBeat’s SpO_2_ measurements in mammalian blood using blue light pulse oximetry. In this experiment, we validated that, as hemoglobin deoxygenates, the transmission of blue light increases and that of red light decreases. This is based on the well-documented absorption and transmission spectrum of hemoglobin [12,48]. We designed this ex vivo experiment to allow us to control blood oxygen saturation over time and capture measurements that would be unhealthy, if not fatal, in human participants. Not only did this experiment allow us to obtain low blood oxygen saturation measurements, it also allowed us to capture continuous change over time, which is not feasible in human-participant trials. In this experiment, laboratory-grade sheep blood was oxygenated by pressurizing it in an oxygen-rich atmosphere. In the human lung, the P_O2_ “increases from approximately 40 mm Hg to 110 mm Hg, a pressure sufficient to ensure at least 95% saturation of hemoglobin with oxygen” [49]. The pressure tank was pressurized to 4137 mmHg (80 pounds of force per square inch) while a peristaltic pump connected to soapstone with a high density of microscopic pores circulated and diffused oxygen-rich air into the blood. This allowed the hemoglobin to bond to the air as it would in the capillaries of the lungs.

**Figure 6:**
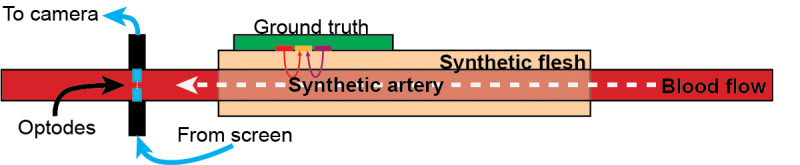
Cross-sectional diagram of the blood oxygen saturation experimental setup.

This setup demonstrated that the change in signal measurements could be attributed to blood oxygen saturation. After being oxygenated as in the previous experiment, sheep blood was pulsed through the artery at 60 beats per minute (bpm) to mimic a heart rate, and the blood was fed back into an open beaker, where it was exposed to standard air pressure and the hemoglobin could continue to deoxygenate. Figure 6 shows how the pulse oximeter was situated on the synthetic skin and artery to measure the ground truth. The experiment was run twice, collecting data continuously for several hours.

### Human-Participant Proof of Concept

To validate that the OptoBeat system would work on human participants, data were collected on three of the authors. Data were collected 3 times for each author. The data collection period was approximately 30 seconds for each sample.

### Ethics Approval

As there was no risk and the participants were all authors of this paper, we did not require Institutional Review Board approval. Data were collected between November 2020 and November 2021.

## Results

The first experiments described in this section demonstrate the effect of skin tone on pulse oximeters. All the measurements in this first experiment are direct current (DC). These DC-component experiments demonstrate how these attributes affect the signal quality when isolated from the much more complex human body.

### Skin Tone Experiment

We measured the transmission of red and IR light in the 3 different skin tones described in the *Methods* section. Experiments were performed using synthetic skin approximately 1 mm thick [46], which has the same physical and optical properties as human skin. By isolating the skin, we removed any potential confounding factors that might further influence the results, such as blood, SpO_2_, skin thickness, or even hydration, among others.

By accessing the photodiode output of a commodity pulse oximeter, the values of the 660-nm (red) and 880-nm (IR) light as it passed through the different samples were recorded. It was important to record these values and not the resulting SpO_2_ values as they had already been fitted to the model. To show that the problem lay in the actual hardware, we compared the red and IR values (Table 1), not the SpO_2_ measurements, as this was where the calculations for SpO_2_ were derived from. In addition, as we used distilled water to ensure that we were only showing the effect of the skin tone (as the SpO_2_ would otherwise change over time), any SpO_2_ measurement would be negligible. The ratios of the resulting 2 bands of light are shown in Table 1. We have also shown the percentage of change in ratios between the different skin tones calculated through a pairwise comparison of the different ratios.

For each skin tone, 2000 samples were collected, and the ratio of IR to red was calculated (type 5: SD 0.42%; type 3: SD 0.25%; type 2: SD 0.25%). We ran an ANOVA test and a pairwise comparison of the results. The results showed that the ratio of transmission for each skin tone was significantly different from the others (*F*_2,5997_=3.12 × 10^5^; *P*<.01), which validated hypothesis 1.

To demonstrate hypothesis 2 (the use of skin tone data in calibrating pulse oximeters), the median sample, classified as type 3, was used as a reference to calibrate the others. For each skin tone, we derived coefficients to normalize the varying ratios between the different skin tones.

The ratio of the ratios was used to produce the following coefficients to normalize the skin tone absorbance of red and IR. The experiment was then replicated with our OptoBeat system using blue and red light, as shown in Table 2. The resulting ratios for hypothesis 2 were as follows (type 3 is always 1 as it was the reference skin tone): 0.8794 (red to IR) and 1.2396 (red to blue) for type 5 and 1.0326 (red to IR) and 1.0340 (red to blue) for type 2.

If it were the case that both wavelengths of light were affected equally, then the only errors that would arise would be due to changes in the signal to noise ratio. However, the change was unilateral, affecting the ratio of the wavelengths and not just the signal strength. Using the ratio of ratios between the alternating current (AC) and DC components of each wavelength did mitigate this to some extent. However, we hypothesized that the AC to DC ratio would also gain error across skin tones owing to their vastly different absorbency characteristics. To confirm this and further validate hypothesis 1, the same experiment as above was conducted using a pump system to move distilled water through a synthetic artery [46] and the 3 separate skin tones. Distilled water was used instead of blood to control for any adulterants, or actual changes in blood oxygen saturation.

As shown in Table 3, the results support the hypothesis that the AC to DC ratio of ratios (Equation 1) would also be affected by skin tone. The 2000 ratio-of-ratio samples showed little deviation from the mean (type 5: mean 0.77%, SD 0.11%; type 3: mean 0.81%, SD 0.11%; type 2: mean 0.94%, SD 0.22%).



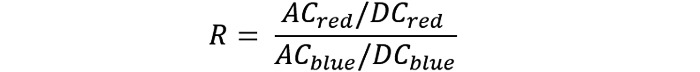



Using an ANOVA test, the results were statistically significant (*F*_2,5997_=8.07 × 10^6^; *P*<.01). Through pairwise multi-comparison, we see that the *R* value changes *by 4.3%* between skin types 5 and 3, *17.7%* between skin types 5 and 2, and *14%* between skin types 3 and 2. As predicted, the ratio of ratios using the AC signal did in fact lower the error; however, it was still significant, proving that, even with the ratio of ratios, hypothesis 1 still held true.

**Table 1:** Results from a direct current pulse oximeter skin tone experiment.

Skin tone type	Infrared value	Red value	Ratio of ratios	Difference from type 5, %	Difference from type 3, %	Difference from type 2, %
5	0.49	0.86	0.58	N/A^a^	12.1	14.8
3	0.51	1	0.51	12.1	N/A	3.2
2	0.50	0.96	0.52	14.8	3.2	N/A

^a^N/A: not applicable (data are compared to themselves).

**Table 2:** Results from a direct current OptoBeat skin tone experiment.

Skin tone type	Blue value	Red value	Ratio of ratios	Difference from type 5, %	Difference from type 3, %	Difference from type 2, %
5	0.8	0.61	0.76	N/A^a^	19.3	16.6
3	1	0.94	0.94	19.3	N/A	3.3
2	0.65	0.59	0.91	16.6	3.3	N/A

^a^N/A: not applicable (data are compared to themselves).

**Table 3:** Results from an alternating current pulse oximeter skin tone experiment.

Skin tone type	Ratio of ratios	Difference from type 5, %	Difference from type 3, %	Difference from type 2, %
5	0.77	N/A^a^	4.3	17.7
3	0.81	4.3	N/A	14
2	0.94	17.7	14	N/A

^a^N/A: not applicable (data are compared to themselves).

### Blood Oxygen Saturation Experiment

**Figure 7:**
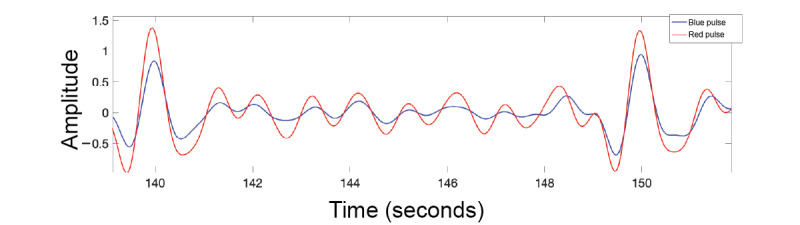
Pulsatile signal of red and blue lights captured in the oxygen saturation experiment.

The data (n=400; 10-second samples, 67 minutes total) were cleaned with a bandpass filter, with cutoff frequencies at 0.5 Hz and 2 Hz (30-120 bpm pass band), and then passed through a Savitzky-Golay filter for further smoothing (third order, 35-sample window). This provided a clear pulsatile signal, as shown in Figure 7. The AC, as measured by the root mean square, and DC (mean) components of the signal were extracted from each signal in 10-second windows (a common window size for this type of calculation) and then inserted into Equation 1 to calculate the ratio of ratios.

**Figure 8:**
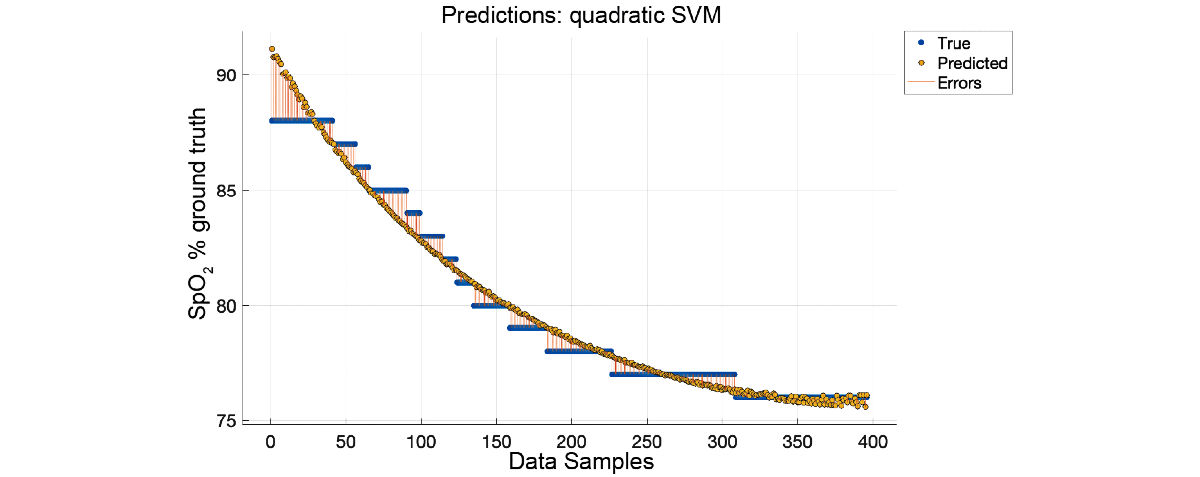
Results of the quadratic support vector machine (SVM) mapping R values captured from OptoBeat to the ground truth. SpO_2_: peripheral blood oxygen saturation.

**Figure 9:**
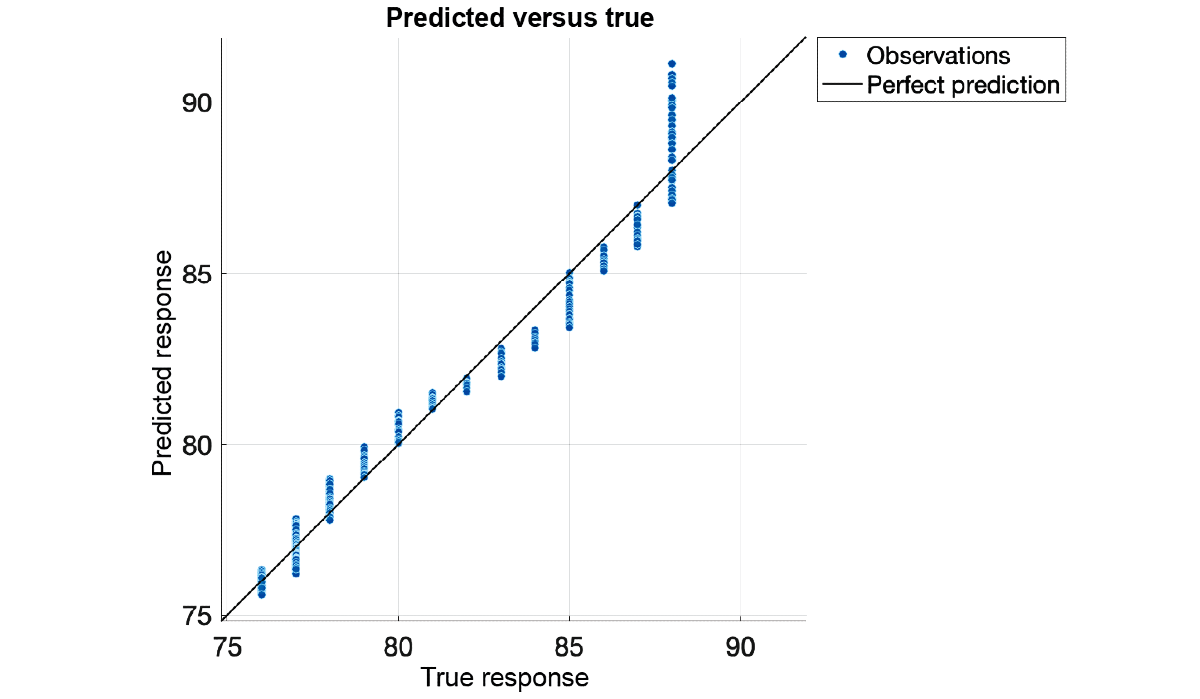
Predicted versus true response from regression.

**Figure 10:**
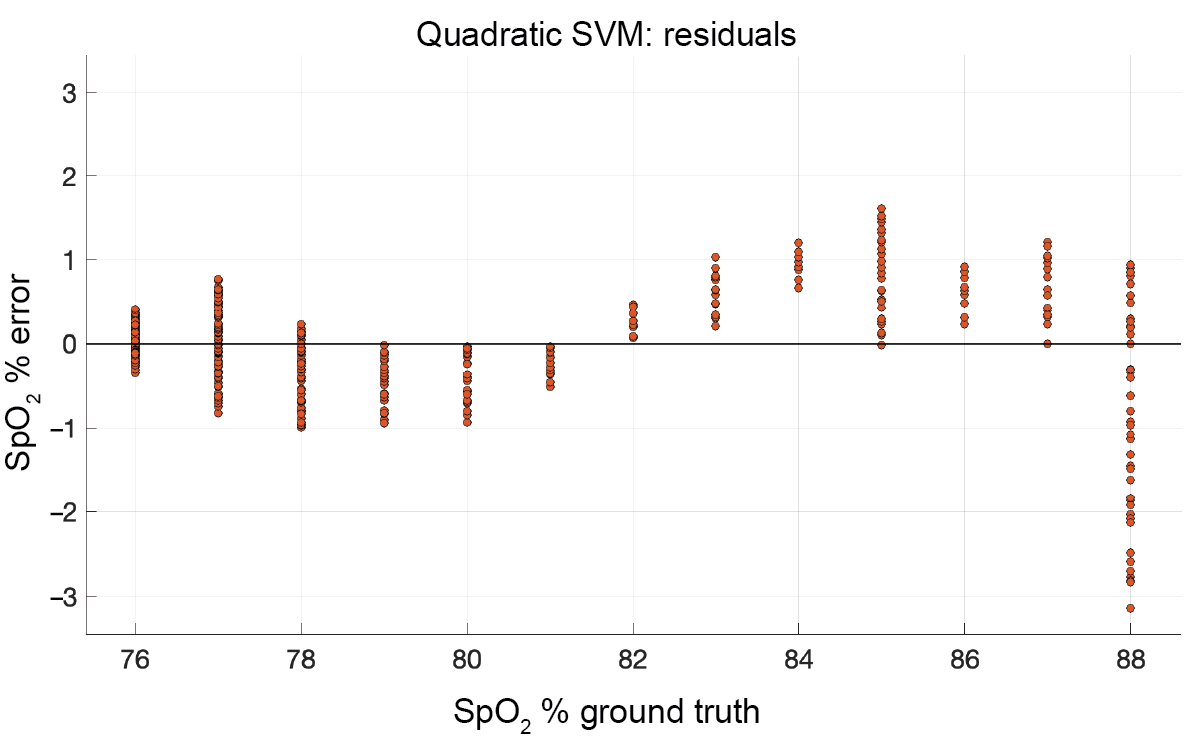
Residual error: OptoBeat and the ground truth. SpO_2_: peripheral blood oxygen saturation; SVM: support vector machine.

The resulting *R* value from Equation 1 has a direct relationship to the SpO_2_ percentage and can be mapped using either a linear equation or regression. A linear mapping of the *R* values to SpO_2_ (*f(x) = mx + b*; m=79.3, b=−5.7 with 95% confidence bounds) rendered very good results (*R*^2^=0.94, root mean square error=1, mean square error=1.1, mean absolute error=0.89); however, we found that a quadratic support vector machine worked better with our data, as shown in Figure 8. This is most likely due to a change in the response curve when more blue light is present with lowering SpO_2_ levels as the blue wavelength is much smaller and does not penetrate the skin as well as red or IR wavelengths. Each yellow dot in Figure 8 is an individual measurement. Each *R* value was calculated over 10 seconds of data. The orange line shows the error from the ground truth for each measurement. The performance, as shown in Figure 9, was validated with a 10-fold cross-validation (*R*^2^=0.97, root mean square error=0.7, mean square error=0.49, mean absolute error=0.5). Figure 10 shows the residual error for each percentage of SpO_2_ measured. The experiment showed that the OptoBeat system measured SpO_2_ as low as *75%* within *–1%* to *+1%* of the ground truth, proving hypothesis 3.

OptoBeat measures continuously with a floating point, whereas the ground truth estimates integer values. We believe that the performance would be more strongly correlated if both were continuous. We plan to evaluate this in the future.

### Human-Participant Proof of Concept

**Figure 11:**
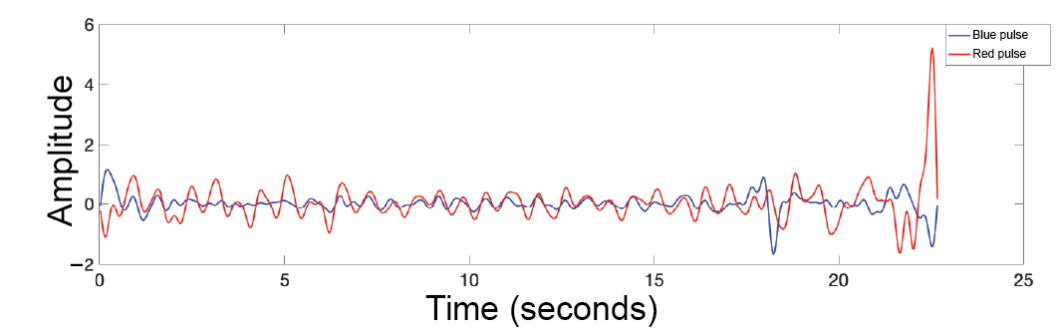
Pulsatile signal of red and blue lights captured in the human-participant proof-of-concept experiment.

Each of the participants (N=3; samples=3 × N, duration=20-30 seconds/sample) measured within –1% to +1% of the ground truth, a commodity pulse oximeter. Figure 11 shows the pulsatile signal captured by OptoBeat. In this sample (23 seconds) from 33% (1/3) of the participants, the heart rate measured by OptoBeat was approximately 111 bpm, and the ground truth was 114 bpm (error=1.7%).

**Figure 12:**
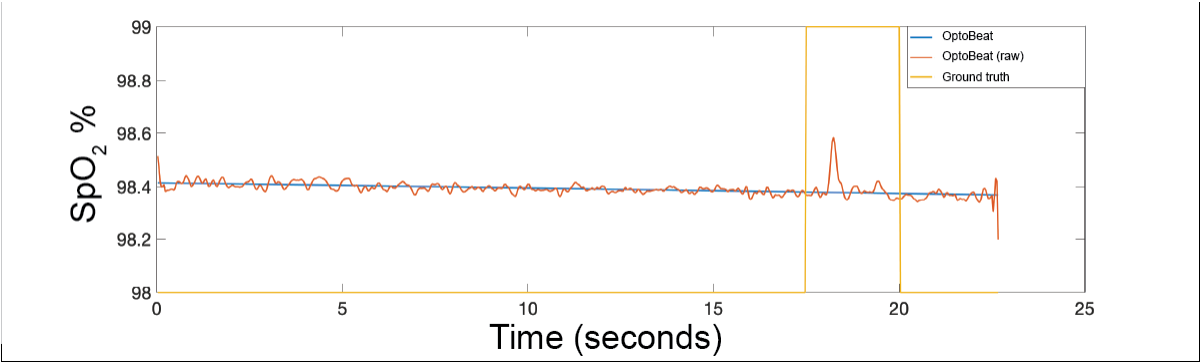
OptoBeat’s peripheral blood oxygen saturation calculations against the ground truth in the human-participant proof-of-concept experiment.

Figure 12 shows the resulting SpO_2_ measurement as captured by OptoBeat from the same data shown on the left. Both the polynomial trend and the raw, individual sample measurements are shown. It can be seen that the ground truth, which is only integer values, is *<0.5%* of the OptoBeat measurement. We believe, as the ground truth is rounded, that this corresponds to the spike (at approximately 17.5 seconds) in the OptoBeat measurement that exceeds *98.5%*. Although each person was healthy and the range of measured blood oxygen saturation was small (between *97*% and *99*% SpO_2_), this demonstrates the feasibility of using OptoBeat in human participants.

## Discussion

### Principal Findings

In this paper, we have presented the design and evaluation of OptoBeat, an optical attachment for smartphones that can reliably measure SpO_2_ and calibrate the measurements according to skin tone via images. The phone-based system for oxygen saturation measurement has potential benefits compared with existing pulse oximeters. With the added benefit of the smartphone’s hardware and computing power, we can not only measure SpO_2_ but also skin tone, which can be used to account for errors in SpO_2_ measurements.

### Strengths and Limitations

Our system is cheaper and simpler to produce than most commercial pulse oximeters. Manufactured at scale, it could be used to shift from a regime of rigid thresholds for admittance or treatment at the point of care to home measurement. At home, trending measures of pulse oximetry could be used to monitor clinical progress or detect silent hypoxemia early [50].

Although this work demonstrates the efficacy of OptoBeat in an ex vivo laboratory experiment, our human-participant proof of concept was only meant to show feasibility, not to validate it for human use. Clinical studies would have to be conducted on a large, diverse population to validate this.

### Future Directions

Moving forward, we plan to deploy and test the current OptoBeat system in a clinical setting. We have partnered with our medical school to take measurements while patients are undergoing cardiothoracic surgery as this will give us access to a range of blood oxygen measurements while a ground truth is being collected without adding additional risk to participants. Furthermore, we plan to redesign traditional pulse oximeters using what we learned from OptoBeat to develop a stand-alone device that can account for variations in skin tone. This will include a large-scale skin tone data collection to build a model that can be leveraged by the device.

### Clinical Implications and Conclusions

Wearable devices currently stand to exacerbate existing health disparities in underserved racial populations who would benefit most from enhanced detection and treatment of health issues. Indeed, such racial disparities occur in the context of more significant cultural issues and reflect the mistrust that many underserved racial populations have for the medical system. This mistrust is primarily due to the medical community’s historical bias toward addressing White Americans’ health needs at the expense of underserved racial populations’ health and well-being. There is an urgent need to overcome the cycle of disparities produced by social injustice across conditions in the places where people live, learn, work, and play. Ensuring that wearables and remote patient monitoring tools are equally efficacious across different populations is necessary to accelerate health equity in the populations who would benefit most from such technology.

**Acknowledgments**

This project was supported by division of Information and Intelligent Systems (RAPID; award number 2031977) of the National Science Foundation. It was also supported by the National Institute on Drug Abuse (grant K23DA041616), the National Institute on Minority Health and Health Disparities (grant P50MD017347), and the National Institute of Allergy and Infectious Diseases (grant P30AI110527) of the National Institutes of Health.

## Appendix

Multimedia Appendix 1 Skin tone calibration equations.

## Abbreviations

AC: alternating current
bpm: beats per minute
DC: direct current
IR: infrared
LED: light-emitting diode
RGB: red, green, and blue
SpO_2_: peripheral blood oxygen saturation
