# Supplementary material for: Equity-Driven Sensing System for Measuring Skin Tone–Calibrated Peripheral Blood Oxygen Saturation (OptoBeat): Development, Design, and Evaluation Study
Source: JMIR Biomed Eng. 2022 Apr 22;7(1):e34934. doi: 10.2196/34934 (PMC11041433; doi:10.2196/34934)
Supplement: Multimedia Appendix 1 [file biomedeng_v7i1e34934_app1.docx]

**Multimedia Appendix 1.** Skin tone calibration equations.

Skin Tone Calibration Equations

Euclidian Distance of RGB images

${distance}_{rgb}=\sqrt{\left( R_{gt}-R_{test} \right)^{2}+ \left( G_{gt}-G_{test} \right)^{2}+\left( B_{gt}-B_{test} \right)^{2}}$

Absolute distance of luminance weighted gray scale images

$${distance}_{gray}= 0.299*\left| R_{gt}-R_{test} \right|+0.578*\left| G_{gt}-G_{test} \right|+0.114*\left| B_{gt}-B_{test} \right|$$
